# Predicted Position Error Triggers Catch-Up Saccades during Sustained Smooth Pursuit

**DOI:** 10.1523/ENEURO.0196-18.2019

**Published:** 2020-01-10

**Authors:** Omri Nachmani, Jonathan Coutinho, Aarlenne Z. Khan, Philippe Lefèvre, Gunnar Blohm

**Affiliations:** 1 Centre for Neuroscience Studies, Queen’s University, Kingston, Ontario, Canada K7L 3N6; 2 VISATTAC, École d’Optométrie, Université de Montréal, Montreal, Ontario, Canada H3T 1P1; 3 Université Catholique de Louvain, Ottignies-Louvain-la-Neuve, Belgium MJ98+V6

**Keywords:** catch-up, eye movements, pursuit, saccades, tracking, trigger

## Abstract

For humans, visual tracking of moving stimuli often triggers catch-up saccades during smooth pursuit. The switch between these continuous and discrete eye movements is a trade-off between tolerating sustained position error (PE) when no saccade is triggered or a transient loss of vision during the saccade due to saccadic suppression. de Brouwer et al. (2002b) demonstrated that catch-up saccades were less likely to occur when the target re-crosses the fovea within 40–180 ms. To date, there is no mechanistic explanation for how the trigger decision is made by the brain. Recently, we proposed a stochastic decision model for saccade triggering during visual tracking (Coutinho et al., 2018) that relies on a probabilistic estimate of predicted PE (PE_pred_). Informed by model predictions, we hypothesized that saccade trigger time length and variability will increase when pre-saccadic predicted errors are small or visual uncertainty is high (e.g., for blurred targets). Data collected from human participants performing a double step-ramp task showed that large pre-saccadic PE_pred_ (>10°) produced short saccade trigger times regardless of the level of uncertainty while saccade trigger times preceded by small PE_pred_ (<10°) significantly increased in length and variability, and more so for blurred targets. Our model also predicted increased signal-dependent noise (SDN) as retinal slip (RS) increases; in our data, this resulted in longer saccade trigger times and more smooth trials without saccades. In summary, our data supports our hypothesized predicted error-based decision process for coordinating saccades during smooth pursuit.

## Significance Statement

The mechanism by which the brain decides when to trigger discrete catch-up saccades during continuous smooth pursuit has eluded researchers for decades. In this study, we present behavioral data complemented by computational model predictions in support of an intuitive trigger mechanism that relies on probabilistic estimation of future position error (PE). Our results add support for a common and shared sensorimotor process for saccades and pursuit. Furthermore, by linking motor control to statistical decision-making, we offer a novel perspective on how sensorimotor prediction and uncertainty modulate oculomotor tracking behavior.

## Introduction

For humans to see an object clearly, its image must fall on a highly specialized region in the retina called the fovea. Foveal tracking of moving objects in the environment is accomplished by smooth pursuit eye movements. However, due to visual feedback delay and sensorimotor noise, the eye can progressively lag behind the target (Krauzlis and Lisberger, 1994a). When significant position error (PE) is accumulated, a catch-up saccade may be triggered to re-foveate the target. Thus, accurate tracking requires a synergistic coordination of saccades and smooth pursuit eye movements to overcome retinal position and velocity mismatches, respectively.

For a long time, it was believed that saccades and smooth pursuit were controlled by independent functional and anatomic systems in the brain (Robinson, 1986). As a consequence, the properties of saccades and smooth pursuit were studied independently. The sensory inputs of pursuit were thought to consist only of velocity and acceleration (Krauzlis and Lisberger, 1994b). Likewise, saccades and their properties were thought to be strictly governed by position inputs (Wurtz and Optican, 1994). However, accurate saccades executed during pursuit challenged this notion, as both position and velocity error must somehow be accounted for in the saccadic system (de Brouwer et al., 2001). Similarly, the smooth pursuit system takes position inputs into account (Blohm et al., 2005; Schütz and Souto, 2011). Consequently, our current understanding describes saccades and pursuit as two outcomes of a synergistic sensorimotor process, sharing sensory inputs, anatomic pathways, and functional regulation (Orban de Xivry and Lefèvre, 2007).

The decision to trigger a catch-up saccade is a trade-off between tolerating a PE when no saccade is triggered or a transient loss of vision during the saccade due to saccadic visual suppression (Schiller, 1986). Thus, when should a saccade be triggered? A double-step ramp paradigm developed by de Brouwer et al. (2002a) allows us to systemically investigate catch-up saccade behavior as a function of position and velocity errors during initiation and maintenance of smooth pursuit. A ramp in target position generates an initial retinal slip (RS), while a step in target position generates an initial PE. de Brouwer et al. (2002b) demonstrated that catch-up saccades were less likely to occur when the expected time-to-foveate the target using pursuit alone [eye-crossing time (T_xe_), -PE/RS] is between 40 and 180 ms into the future, which was referred to as the smooth zone. However, T_xe_ is a deterministic measure, providing no mechanistic explanation for the trigger mechanism or how to account for sensory or motor uncertainty. Similarly, early models describe the trigger decision for a stationary saccade as a linear rise to threshold using the LATER model but do not address sensorimotor context of the target or ongoing smooth pursuit (Carpenter and Williams, 1995; Reddi and Carpenter, 2000). As such, a re-examination of the saccade trigger is needed in this new context.

**Figure 1.**
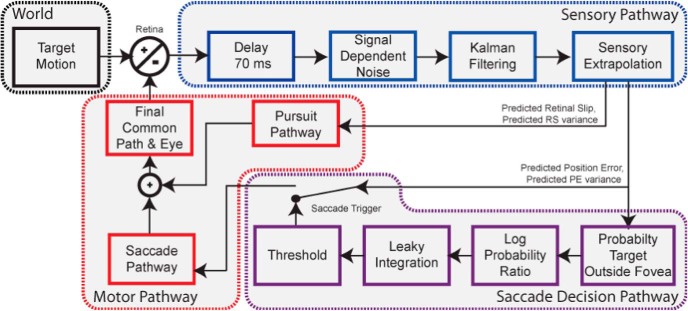
Saccade trigger model overview. A block diagram overview of the Bayesian decision model for saccade triggering. The sensory pathway estimates noisy signals from the world and extrapolates those to predict future errors. The decision pathway estimates confidence in future error prediction and triggers a saccade for a certain confidence threshold. The motor pathway adds the pursuit and saccade motor commands. Adapted with permission from Coutinho et al. (2018).

We have recently developed a Bayesian decision model that offers a mechanistic explanation of the catch-up saccade trigger (Fig. 1).

Our model relies on probabilistic estimation of predicted PE (PE_pred_) through Kalman filtering, i.e., an estimate with uncertainty due to sensory noise. This probabilistic estimate of PE is transformed into an estimate of confidence that a target is outside the fovea. Analogous to the evidence accumulation of a LATER model, confidence rises as sensory evidence is accumulated, rising faster with larger estimates of PE_pred_ and/or lower uncertainty. Thus, the occurrence and time to trigger of the saccade is determined if and when this confidence estimate reaches threshold for saccade trigger.

Our current study aims to answer how PE and RS inputs are used by human subjects to make a trigger decision. The classical index thought to trigger catch-up saccades is a deterministic T_xe_. Throughout the study, we compare the explanatory power of T_xe_ to our probabilistic trigger mechanism based on future error. We hypothesized that the saccade trigger mechanism is an uncertainty dependent decision-making process that utilizes prediction of future sensory inputs. Using a wide range of moving target parameters to induce catch-up saccades during steady-state pursuit, we will be testing the following novel qualitative predictions that our model makes (rationale in the analysis plan): (1) large pre-saccadic PE_pred_s from target motion lead to less variable and shorter saccade trigger times; (2) target step followed by motion towards the fovea will produce highly variable as well as long saccades trigger times, whereas motion in the opposite direction will produce low variability and short saccade trigger times; (3) applying a Gaussian blur to the target will increase uncertainty in PE_pred_ and thus increase variability in saccade trigger times; and (4) for a given crossing time, large velocity steps (VSs) accrue larger PEs and thus lead to more occurrences of catch-up saccades with short trigger times. Null hypothesis: saccade triggering is determined by T_xe_, independently of PE_pred_s, where negative or large positive crossing times trigger saccades.

## Materials and Methods

This study is a quantitative analysis study. Eye movement data are collected from subjects and analyzed to reveal the presence or absence of predicted patterns of behavior (see above predictions). No experimental blinding was involved in this study since eye movement control is stereotyped and catch-up saccade triggering appears to be automatic, often unconscious, and therefore beyond cognitive control. There was no existing data in our lab pertaining to smooth pursuit catch-up saccade triggering. While our study produced similar data to de Brouwer’s catch-up saccade study, this dataset was recorded with modern video eye-tracking techniques, involved new experimental conditions and designs, and the analysis was guided by our model to uncover the functional mechanism leading to saccade triggering (as opposed to de Brouwer’s purely descriptive analysis).

### Pre-registration

This study was pre-registered at *eNeuro* as a registered report. The purpose of registered reports is to minimize biases in deductive science by pre-registering the proposed hypotheses of the experiment. The hypotheses, methods, and analysis plan of a registered report are reviewed before the research being conducted to ensure sound scientific reasoning. Another goal of pre-registration is to promote open science. As such, the registered report is available on the open-science framework (OSF), the code necessary to run and analyze the experiment is available on GitHub, and the raw and labeled dataset is available on Dryad, links provided. Registered report OSF link: https://osf.io/wvjbf/; task and analysis code GitHub link: https://github.com/BlohmLab/SaccadeTrigger; data repository Dryad link: https://doi.org/10.5061/dryad.245j1p8.

### Sampling plan

This study focused on automatic, stereotyped behavior and all participants exhibited similar patterns of behavior. A power analysis based on effect sizes from the model concluded that the minimum number of participants need is *N* = 12. We recruited 15 participants (seven female) from the neuroscience department who were unfamiliar with the double-step ramp task. Eligible subjects had normal or corrected-to-normal vision. Each participant completed 10 data collection sessions lasting ∼45 min with 10 blocks each consisting of 50 trials per block, yielding a total of 5000 trials per participant and 75,000 trials altogether.

### Experimental procedure

A double step-ramp task (de Brouwer et al., 2002a) generated with MATLAB (MathWorks, Inc) using the Psychophysics Toolbox (Brainard, 1997) was displayed on a ViewPixx (VPixx Technologies, 120-Hz refresh rate, resolution 1920 × 1200) screen positioned 50 cm from the participants spanning 60° of their visual field. All trials began with an initial fixation target (0.5°, black) positioned 20° to the left or right of the visual field followed by an abrupt position step (PS) away from the center (2°/4°/6°) with consecutive VS (10°/20°/30°/s) toward the center to induce steady-state smooth pursuit. After a variable time-period (500–700 ms), a second step-ramp occurred in either direction. The VS was randomly chosen from a uniform distribution ranging from –50°/s to 50°/s. PSs were selected (–20° to 20°) based on the VSs to limit the absolute time-to-foveate (-PS/VS) to under 1000 ms. Target velocities and displacements varied randomly between each step-ramp and trial which ensured that the target jumps remained unpredictable. Each recording session alternated between clear and blurred target conditions. The purpose of the blurred target condition was to observe the influence of additional sensory noise on behavior. In the blurred target condition, the target was a 2D Gaussian blur (50°, σ = 5°). Half of the participants began with the blurred condition and half with the clear condition. Eye movements were tracked using an EyeLink (SR Research EyeLink 1000) video-based eye tracker with a sampling rate of 1000 Hz. Position signals were low-pass filtered by a zero-phase digital filter (cutoff: 50 Hz). Velocity and acceleration were derived from position signals using a central difference algorithm and saccades were detected using an acceleration threshold of 750°/s^2^. All data of the target and eye movement trajectories and timing was stored on the computer during the experiment to be used for offline analysis. To minimize measurement error, participants performed calibration and validation tasks on blocks 1, 4, and 7. Data collection began only if the calibration error obtained from EyeLink was below 0.2 on the *x*-axis relevant to the task.

### Pre-processing

Individual trials were visually inspected for errors. Trials were discarded from the analysis if participants clearly did not track the target (e.g., due to a distraction), if blinks occurred during or after the second double-step (our critical period), if eye tracking data were missing (e.g., pupil detection failed), or if a saccade occurred during the second double-step (because visual processing would be disrupted). The remaining trials were filtered to remove occurrences of double saccades, as their execution and main sequence differed from the rest of the data, and saccades occurring after the second target step but made to the trajectory of the first target ramp. The total percentage of trials discarded was 23%, yielding ∼58,000 trials for analysis.

### Computations on data

We computed instantaneous PE_pred_ over time as follows: PE_pred_,

(1) $$PE_{pred}(t)=PE(t)+dt*RS(t),$$

where PE is the difference between target and eye positions (target-eye) and RS is the difference between target and eye velocities (target-eye). The variable dt is the extrapolation duration and is typically between 90 and 290 ms (Robinson, 1986; de Brouwer et al., 2002b). By default, we used dt = 150 ms to extrapolate PE as used by our model. Our model predicted that a saccade will be triggered if PE_pred_ is significantly different from zero, assuming a certain measurement noise.

Target-crossing time (T_xt_) is a measure of how much time it will take for a target to re-cross its initial trajectory following a step in position and velocity. This measure is agnostic to any behavioral measurements as it only relates to target motion dynamics. T_xe_, on the other hand, measures the time it will take for the eye to contact the target given the current position and velocity error. At the moment of target step, T_xt_ and eye-crossing are comparable in magnitude. T_xt_ and T_xe_ were computed as follows: T_xt_,

(2) Txt=−PSVS,

and T_xe_,

(3) Txe=−PERS,

where PS is position step, VS is velocity step, PE is position error, and RS is retinal slip.

**Figure 2.**
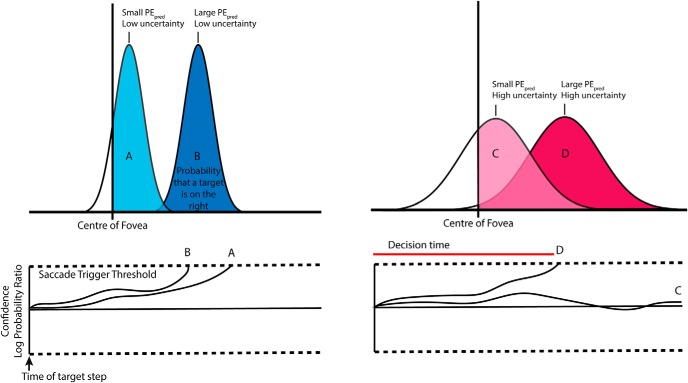
Model illustration of saccade trigger decisions. A representation of the interaction between PE magnitude, uncertainty, and the confidence that the target is outside the fovea. The confidence that the target is to the right of the fovea is represented by the ratio of the AUC to the right of fovea and the left of the fovea (Fig. 2, top panels). Saccades are triggered when the confidence signal rises to threshold (Fig. 2, bottom panel).

The trigger mechanism in our model relies on confidence, i.e., the probability that the target is outside the fovea [area under the curve (AUC) in Fig. 2, top panel].

Incoming sensory input is noisy and has a level of uncertainty that depends on the sensorimotor context (SD in Fig. 2). Thus, when PE is small (Fig. 2*A*,*C*, top panel), higher uncertainty can considerably reduce the confidence that the target is to the right of the fovea. When the magnitude of PE is high (Fig. 2*B*,*D*), variations in uncertainty do not significantly affect the confidence that the target is to the right of the fovea. When this confidence reaches a threshold (Fig. 2*A*,*B*,*D*, bottom panel), a catch-up saccade is triggered to the peak of the probability density function. The confidence signal for lower PEs with high uncertainty fails to reach threshold and will not trigger a saccade (Fig. 2*C*, bottom panel). The decision threshold is a tuned model parameter describing the amount of accumulated confidence necessary to trigger saccades.

**Figure 3.**
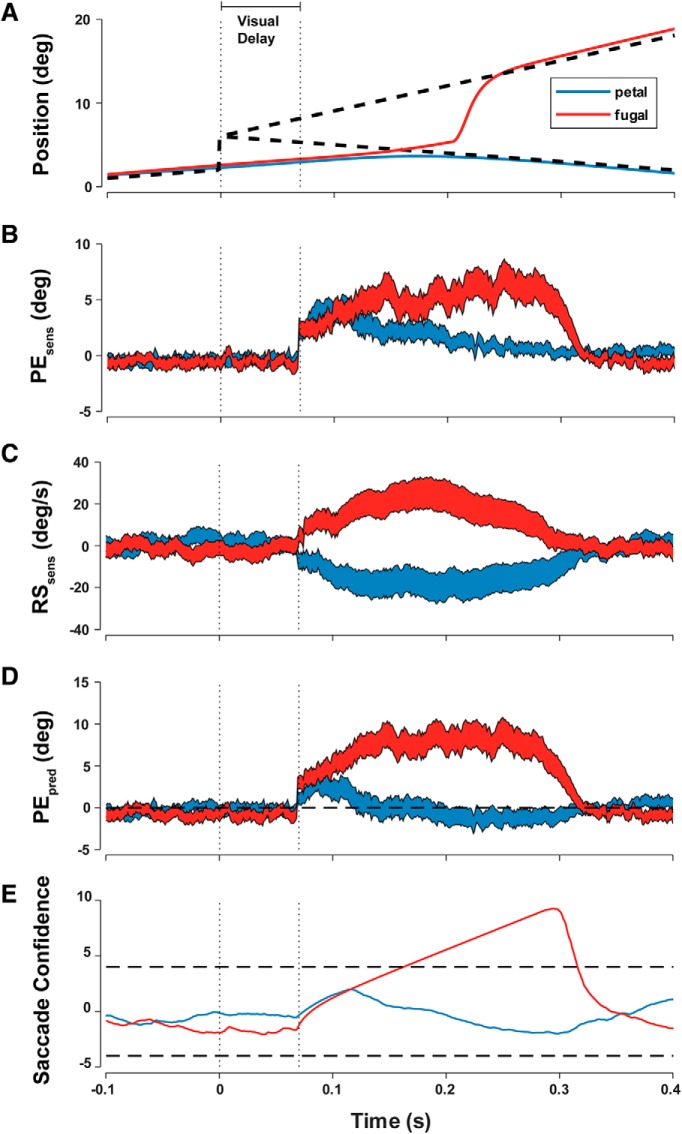
Single trial model simulations. ***A***, Target motion that results in a saccade and another that results in smooth pursuit with no saccade. ***B***, ***C***, Noisy PE and RS sensory signals are estimated continuously using a Kalman filter. ***D***, Future PE is continuously estimated using sensory signals. ***E***, A rise to threshold using a log probability ratio or confidence of sensory evidence leads to a trigger or no trigger decision.

Model simulations of two step-ramp trials demonstrate the evolution of sensory estimates as evidence accumulates and the variance from the Kalman filter decreases (Fig. 3).

The rate of rise to threshold determines the saccade trigger time. “Red” trials show large and increasing predicted errors with low uncertainty and thus the rate of rise to trigger saccades is higher. In the “blue” trial, confidence in predicted error never reaches threshold and a saccade is not triggered (Fig. 3).

Small but sustained predicted errors with low uncertainty will produce a slower rise to threshold and ultimately trigger a late saccade. If the predicted error is very large, uncertainty will have little influence on the rate of rise and a saccade will be triggered quickly.

### Pre-registered hypotheses

#### Hypothesis 1

Small pre-saccadic PE_pred_s from target motion lead to highly variable and longer saccadic trigger times.

To test this hypothesis, PE_pred_ was calculated continuously (Eq. 1) for all trials for each individual participant. Trials were then sorted into saccade trials and smooth trials based on whether a saccade occurred within 400 ms of the second target step. For saccade trials, PE_pred_ for each trial was averaged over a 50-ms window from target step. Trials were then sorted into 5° bins of increasing magnitude in PE_pred_ with the corresponding saccade trigger time (saccade onset time – target step time) for each trial. We then calculated the saccade trigger time median and interquartile range (IQR) for each bin. Median and IQR were compared across participants using a repeated measures one-way ANOVA analysis. To further support our hypothesis and avoid commonly encountered issues with the frequentist approach to analysis, data were also examined by estimating the Bayes Factor (Wagenmakers, 2007). We expected a significant difference between saccade trigger time medians and variability in the low versus high PE_pred_s.

#### Hypothesis 2

Target step followed by motion toward the fovea (foveopetal) will produce highly variable and longer saccade trigger times, whereas motion in the opposite direction will produce low variability and short saccade trigger times.

**Figure 4.**
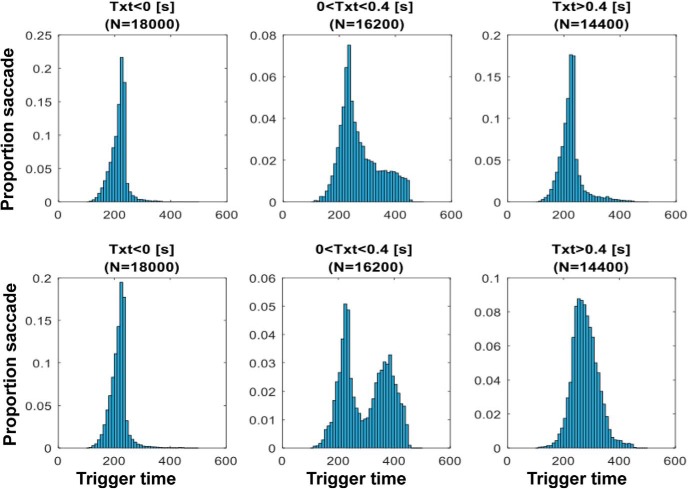
Model simulations of trigger time distributions. Model generated saccades trigger time distributions shown made to a clear target (Fig. 4, top panels) and a blurred target (Fig. 4, bottom panels). When the target is moving away from the fovea (T_xt_ < 0), uncertainty in PE does not significantly change trigger time distributions. For T_xt_>0, increasing uncertainty in PE using blurred target increases saccade variability, especially around the smooth zone (0 < T_xt_ < 400).

The direction of the position or VS can also significantly influence saccade trigger times. For example, using PE_pred_ (Eq. 2), a PS of 5° and VS of 10°/s produces a PE_pred_ of 6.5° while the same PS with a VS of –10°/s produced a PE_pred_ of 3.5°. Both PEs may require a saccade, however, in the former the target is continuously moving away from the fovea, while in the latter the PE_pred_ is decreasing. We can further capture this by calculating the T_xt_ for target motion (Eq. 1). In the former situation, T_xt_ = –500 ms, negative, which requires an immediate saccade as the target’s distance from the fovea is increasing, whereas in the latter, T_xt_ = 500 ms, positive, the decision to trigger a saccade is not as obvious as the target is approaching the eye and a participant may wait to accumulate more evidence. We expect to see more variability in trigger times in saccades occurring in the positive T_xt_ range and more narrow trigger time distributions in the negative T_xt_ range (Fig. 4, top panels).

To test this, T_xt_ was calculated (Eq. 1) for all trials for individual participants and the corresponding saccade trigger time (target step time – saccade onset time) for that trial was obtained. Trials of individual participants were then sorted into three bins according to their T_xt_ (T_xt_ < 0, 0 < T_xt_ < 400, T_xt_ > 400). Individual participant’s trigger time distributions for each bin were calculated and compared across participants using a repeated measures one-way ANOVA with T_xt_ bin as a factor. *Post hoc* comparisons were performed using paired *t* tests with familywise corrected error rates. We then calculated the Bayes factor using the sum of squares from the ANOVA analysis. We expected to see narrow trigger time distributions for negative T_xt_ and wider trigger time distributions for positive and large T_xt_.

#### Hypothesis 3

Applying a Gaussian blur to the target will increase uncertainty in PE_pred_ and thus increase variability in saccade trigger times.

We hypothesized that increase in uncertainty of PE_pred_ due to a blurry target can lead to higher variability of saccade trigger times (Fig. 4). To test this, we compared trials in the clear condition with those in the blur condition. We again obtained trigger time distributions for the two conditions and compared the median and IQR of saccades in the PE_pred_ and three T_xt_ using a two-way ANOVA analysis and additional Bayes factor estimation. We expected to see more variability in the blurred target condition than in the clear target condition

#### Hypothesis 4

For a given T_xt_, large VSs accrue larger PEs and thus lead to more occurrences of catch-up saccades with shorter and less variable trigger times.

**Figure 5.**
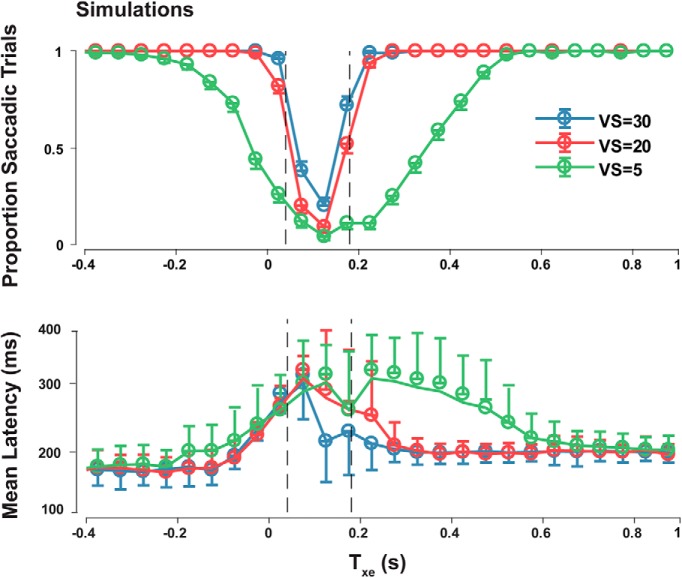
Model simulation trends of saccade trial proportions and trigger times. Model simulations of proportion of saccade trials and the trigger times of saccade trials compared to T_xe_ (-PE/RS) for different VSs. The smooth zone boundaries from de Brouwer et al. (2002b) are marked by vertical dashed lines. The proportion of saccades near the smooth zone increases with increasing target VS. The saccade trigger times decrease with increasing target VS.

Our model predicted that contrary to de Brouwer’s findings, the smooth zone is not static but rather decreases in size with increasing VS. At higher VSs, pursuit velocity is typically below target velocity. This may lead to larger PEs and therefore larger PE_pred_s and more saccades (Fig. 5, top panel). Our model also predicted that target VS size will significantly change the trigger time distributions (Fig. 5, bottom panel)

We compared trials where saccades occurred within 400 ms of target step and those where no saccades were observed in that time window. All trials were sorted into five equally spaced bins according to their absolute second VS (0–50°/s). Trials were then sorted once more according to their T_xt_ as in the previous analysis. We then used two-way ANOVA to compare the proportion of saccade trials and their trigger time in three VS conditions (0–10°/s, 20–30°/s, 40–50°/s) and each T_xt_ bin to observe whether the increase in RS led to a narrower smooth zone, shorter saccade trigger times, and narrower distributions. We estimated the Bayes factor and performed *post hoc* comparisons using paired *t* tests with familywise corrected error rates.

#### Null hypothesis

Saccades are triggered when time-to-foveation (-PE/RS) is smaller than 40 ms or larger than 180 ms as described by de Brouwer et al., 2002b.

For reproducibility, we compared our analyzed data collected from our “clear” condition to the analysis from de Brouwer et al., 2002b.

For all analyses, we concluded that differences in saccade trigger times are significant if the unrounded *p* value from our ANOVA analysis (with conservative Bonferroni correction for multiple comparisons) was smaller than 0.05.

## Results

**Figure 6.**
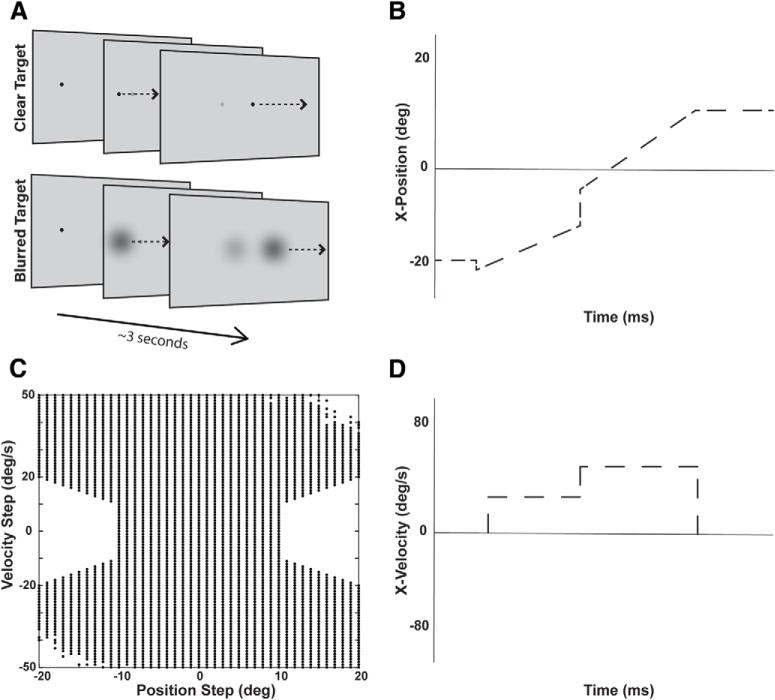
Double step-ramp task. ***A***, Representation of the double step ramp task on a screen. Each participant completed ten alternating sessions of clear and blurred target conditions. Each session consisted of 10 blocks of 50 trials each. ***C***, Parameter range for PSs and VSs used to induce catch-up saccade triggering. Large steps were omitted as they resulted in off-screen targets. ***B***, ***D***, Position, velocity-time graphs of the tracking dot for a typical trial. Shown are a positive PS (to the right) and a positive VS (rightward acceleration).

Our study aimed to recast former proposals for the triggering of catch-up saccades. The first section of our results (Figs. 6-Figs. 8) reintroduces the double-step ramp paradigm and sensory phase plots as a means to explore overall behavioral trends of catch-up saccades, with a novel addition of a side-by-side comparison of typical saccade trials and their corresponding temporal evolution on the phase plot. The purpose of this section is to highlight the similarities in behavioral trends of saccade triggering to previous work while also pointing out some explanatory gaps that motivated this current study. Next, we demonstrate how PE_pred_ satisfactorily explains both the occurrence of catch-up saccades and the saccade trigger time (Figs. 9, 10). Data presented in the figures provide comparisons between clear and blurred target conditions to observe the effects of sensory uncertainty. Additionally, all data are computed using both PE_pred_ and the previously proposed T_xe_ or T_xt_. These comparisons intend to demonstrate the superior explanatory power of PE_pred_ computation in the trigger mechanism. We then present trigger time distributions, summary bar graphs, and statistics in support of our pre-registered hypotheses (Figs. 11, 12). We conclude by exploring an interesting finding from the trigger time distributions (Figs. 13, 14) and provide final thoughts on the nature of the saccade trigger mechanism (Fig. 15).

### Temporal evolution of sensory inputs

The trigger system for catch-up saccades depends on position and velocity sensory inputs. As such, in this section we explore how varying these inputs changes saccade and smooth pursuit behavior. The step-ramp paradigm had been widely used in behavioral eye movement studies as a tool to induce smooth pursuit tracking with minimal catch-up saccades. As subjects fixate on a foveal target, the target abruptly steps peripherally by two to six visual degrees while simultaneously moving toward and past its previous location. If the target re-crosses the fovea in a short amount of time (100–200 ms), the eye responds by slowly accelerating in the same direction as target movement, rather than triggering a saccade to the peripheral target location. During steady state pursuit of the target, visual delays and imperfect gain can lead to the eye gradually falling behind the target, resulting in RS, a mismatch of target and eye velocity. Consequently, RS is accompanied by PEs, a mismatch between target and eye position. If sustained and large enough, both PE and RS lead to a catch-up saccade. To investigate under which conditions a catch-up saccade is triggered during sustained pursuit, we introduced a second step in both target position and velocity with a wide range of values (Fig. 6).

**Figure 7.**
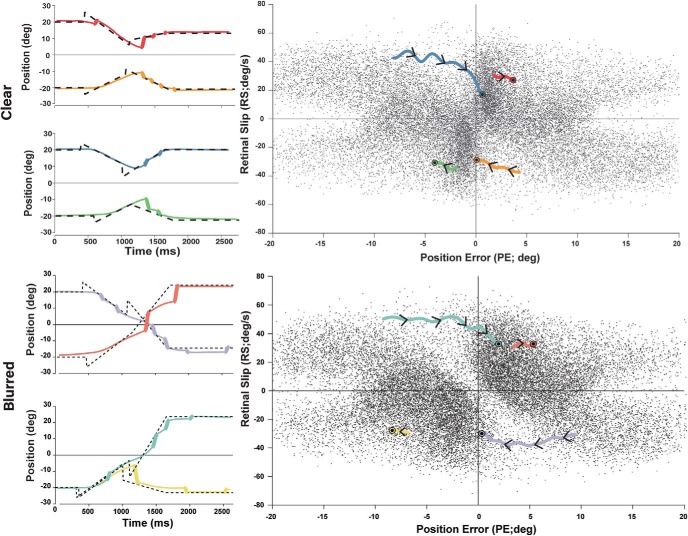
Sensory phase plot and typical saccade trials. For each saccade trial, PE and RS were sampled 100 ms before saccade onset and plotted on a phase plot. Left, Four typical trials for clear and blurred target conditions plotted in pairs on a position-time graph, with saccades in bold. Right, PE and RS phase plots of all trials for clear and blurred target conditions. The colored traces correspond to the evolution of sensory inputs of the typical trials on the left from the target step to 100 ms before saccade onset (represented by black circles). Note that when PE and RS have opposite signs, PE naturally decreases and when PE and RS have the same sign, PE naturally increases in absolute value.

Following this additional random step in target position or velocity, the evolution of PE or RS are highly dependent on the motion dynamics of the target and pursuit ability of the subject. For example, when a PS occurred in the positive (rightward) direction and a VSs occurred in the negative (leftward) direction, or vice versa, PE naturally decreased as the target crossed its original trajectory. Conversely, if both position and VSs occurred in the same direction, PE continued to increase from the original PS. These dynamics are further explored in the typical trials in Figure 7, left panels.

In a typical trial, a subject may trigger several saccades to track the target. Of interest is the first saccade triggered after the second target step, since the system was perturbed with specific and controlled position and VSs. The range of behaviors observed can be classified into three categories: early saccades, late saccades, and smooth trials where no saccade occurred for the first 400 ms (de Brouwer et al., 2002b). In Figure 7, left panels, late saccades such as blue and “turquoise” often result from a failure to estimate or match target velocity, as the eye anticipates crossing but eventually moves past the clear target. Importantly, in the blurred condition, subjects demonstrate comparable foveal tracking during sustained pursuit. Clear red, “green,” blurred “yellow,” and “pink” represent targets moving foveofugally (away from fovea) after the target step, whereas the rest of the trials move in the foveopetal (toward fovea) direction.

To observe overall behavioral trends of saccade occurrences under different sensory conditions, we plotted a sensory phase plot (Fig. 7, right panels) of the instantaneous PEs and RSs 100 ms before saccade onset, thought to be the last instant that sensory information can influence saccade decisions to provide enough time for saccade programming and execution (Orban de Xivry and Lefèvre, 2007). The double coned region with a visibly lower density of saccade occurrence is referred to as the smooth zone (de Brouwer et al., 2002b). This region is more evident under the blurred condition, as higher uncertainty led to more smooth trials. The ratio of PE and RS that defines the slope boundaries of this zone is defined as the T_xe_ from 40 to 180 ms, although it is immediately evident that many saccades are still triggered within the smooth zone. Furthermore, of interest to us was how such sensory inputs evolve temporally from the moment of target step, depicted by the overlaid colored traces. Two high density regions of saccades are apparent on either side of the “0 PE.” These regions result from late saccades such as “orange” and blue that move into the smooth zone but fail to match target velocity and thus trigger a saccade as soon as they cross the target. Short saccade trigger times such as red or green tend to start far from and continue to move away from the smooth zone.

**Figure 8.**
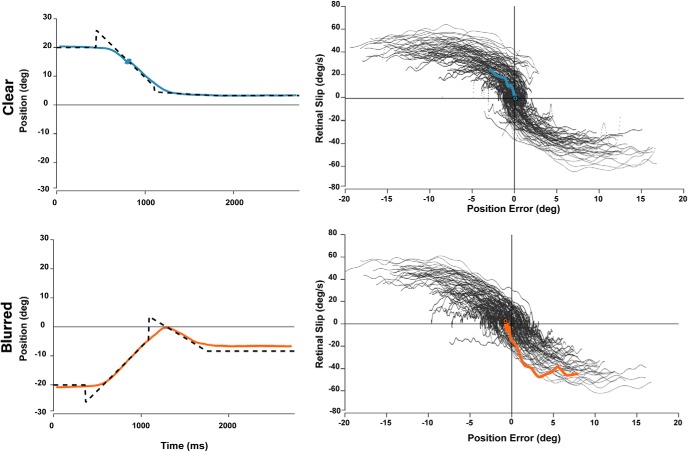
Sensory phase plot and typical smooth trials. For smooth trials where no saccade occurred within 400 ms of target step, PE and RS were sampled continuously and plotted on a phase plot. Left, Typical smooth trial on a position-time graph where eye traces are colored. Trials are considered smooth if no saccades occur within the first 400 ms after target step. Right, Phase plot showing continuous sampling of PE and RS for a subset of trials. Smooth trials tend to begin in the upper left or lower right corners where PE naturally decays as PE and RS are in opposite directions. The colored traces correspond to the typical trials on the left, showing the evolution of PE and RS toward the center where near-perfect tracking is achieved. The red dotted traces represent the smooth zone boundaries from de Brouwer et al. (2002b).

Figure 8 shows continuous sampling of PE and RS for trials where no saccades occurred in the first 400 ms, classified as smooth trials. Successful smooth trials result when participants accelerate or decelerate to match target velocity and minimize both PE and RS to ∼0. This region of dense smooth trials corresponds to the low saccade density zone in the Figure 7.

Together, the traces in Figures 7, 8 provide intuition for how sensory inputs evolve through time and lead to a saccade trigger, although on their own, they are only descriptive of behavioral correlates. Moreover, the smooth zone boundaries and the corollary T_xe_ index poorly explain several trends in the data. For example, some trials that either briefly cross or even remain in the smooth zone nevertheless trigger a saccade. Conversely, many smooth trials begin far outside the smooth zone and proceed toward it. The variability in behavioral observations contradicts previously established notions of an absolute decision criteria of saccade trigger based on T_xe_ and suggests a probabilistic explanation driven by statistical decision theory. In this view, noisy sensory estimates of PE and RS are continuously sampled and used to make predictions of future PE. In turn, these predictions are used to make motor decisions to trigger or not to trigger a catch-up saccade. Importantly, high uncertainty in the prediction will delay or suppress saccade triggering.

### PE_pred_ explains saccade occurrence

**Figure 9.**
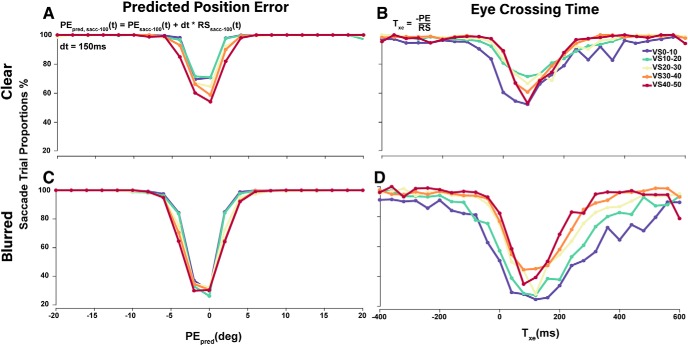
Proportion of saccade trials relative to PE_pred_ and T_xe_. PE_pred_, PE, and RS were sampled ∼100 ms before saccade onset for saccadic trials and averaged over the first 400 ms after target step for smooth pursuit trials. ***A***, ***C***, Saccade trial proportions plotted for a given PE_pred_ from –20° to 20° in bins of 2°. ***B***, ***D***, Saccade trial proportions plotted for a given T_xe_ from –400 to 600 ms in bins of 50 ms.

We hypothesized that any decision signal that leads to a catch-up saccade must reach a decision threshold ∼100 ms before saccade onset, to allow time for saccade preparation and motor execution. Therefore, we sampled PE and RS 100 ms before the first catch-up saccade of each saccade trial and computed PE_pred_ using Equation 1, as well as T_xe_ using Equation 3. For smooth trials, we averaged PE and RS over the initial 400 ms following second target step. In Figure 9, saccade trial proportions are plotted as a function of both PE_pred_ and eye crossing time for different magnitudes of VSs.

The large dip in saccade occurrence from centered at 0 ± 5° for PE_pred_ and 100 ±100 ms for T_xe_ has been referred to as the smooth zone. The boundaries of the smooth zone are far more sensitive to differences in VS when plotted by T_xe_ compared to PE_pred_, likely due to the instability of the T_xe_ signal as velocity errors approach zero, as RS is in the denominator. Figure 9*A*,*C* shows near 100% saccade trials outside the –5° to 5° range, consistent with the hypothesis that PE_pred_ plays a central role in the saccade trigger decision. As anticipated (Fig. 9*A*) larger VSs lead to higher signal-dependent noise (SDN) and thus lower proportion of saccades. Interestingly, higher sensory uncertainty under the blurred target condition noticeably reduces the influence of VS related SDN and also significantly reduces saccade proportions, suggesting that noise from a blurred target is a larger driver of uncertainty and strongly modulates motor decisions.

### PE_pred_ and uncertainty modulate saccade trigger time

**Figure 10.**
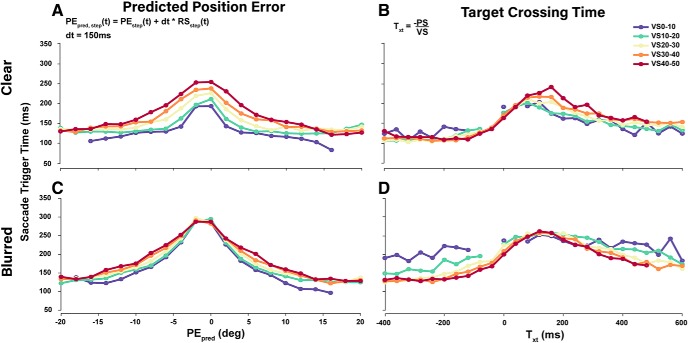
Collapsed saccade trigger time for ranges of PE_pred_ and T_xt_. Collapsed trigger time medians were plotted against PE_pred_ and T_xt_ for the relevant parameter ranges and increasing VSs. ***A***, ***C***, Collapsed Saccade trigger times as a function of PE_pred_ for different VSs. ***B***, ***D***, Collapsed saccadic trigger times as a function of T_xt_ for different VSs showing an increase around values corresponding to the smooth zone. Missing data points for VS0-10 and VS10-20 result from PS being drawn from a uniform discrete distribution.

Further exploring how well PE_pred_ explains the trigger decision, we investigated the effects of PE_pred_ on trigger time, which was defined as the time from target step to saccade onset. To make inferences on how the magnitude of PE_pred_ influences trigger time, PE_pred_ was sampled at target step and averaged over 50 ms from target step. Similarly, at the time of target step, T_xt_ (Eq. 2) was used in place of T_xe_ in accordance with previous work. Figure 10*A*,*C* shows that trigger times increase as PE_pred_ approaches zero, and comparably higher VS lead to longer trigger times due to higher SDN.

Under high uncertainty conditions (Fig. 10*C*) trigger times increase considerably only for small PE_pred_ but remain the same for very large PE_pred_, supporting our model predictions (Fig. 2). Notably, in Figure 10*B*,*D*, although the T_xt_ plots show similar patterns of longer trigger times surrounding the smooth zone, the trends are far noisier at lower VSs and large positive T_xt_. Under pre-registered hypothesis 4, we predicted longer and more variable trigger times for smaller VSs for a given T_xt_. A two-way ANOVA revealed a significant interaction between VS and T_xt_ medians (*F*_(2,14)_ = 5.791, η^2^_p_ = 0.293, *p* = 0.001, BF_10_ = 1.001e+35) but no significant interaction of IQRs (*F*_(2,14)_ = 1.064, η^2^_p_ = 0.071, *p* = 0.314, BF_10_ = 2.846e+20) in the clear target conditions. However, *post hoc* analysis revealed that smaller VSs lead to shorter and less variable trigger times only when T_xt_ was positive (*p* = 0.009), opposite to our prediction. This trend does not hold under blurred conditions as seen in Figure 10*D*. We conclude that although T_xt_ influences trigger times, its effects were not predictable and consistent across different experimental conditions.

### Pre-registered hypothesis 1 and 3

**Figure 11.**
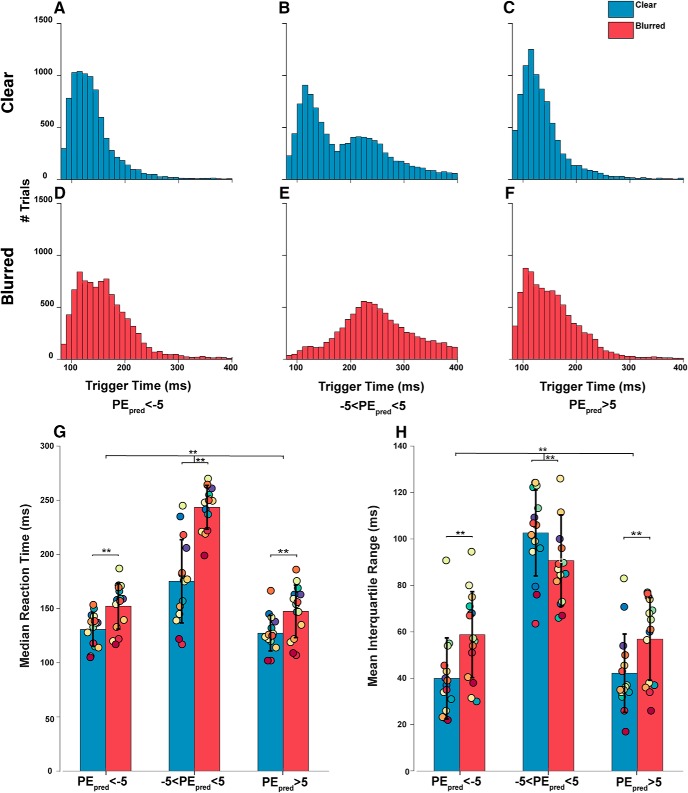
Saccade trigger time distributions and medians by PE_pred_. Saccade trigger times are measured from target step to saccade onset. to make conclusions on how PE_pred_ contributes to saccade trigger time, PE_pred_ here was also sampled from target step over 50 ms and averaged, rather than ∼100 ms before saccade onset. Trials were grouped into bins of large negative (***A***, ***D***), small (***B***, ***E***), and large positive (***C***, ***F***) PE_pred_. ***G***, Collapsed median trigger times for all subjects for clear and blurred condition. ***H***, Collapsed IQR of all subjects for clear and blurred condition; ** represents a significant difference between groups with a *p* < 0.001. Each colored dot represents a particular subject’s mean value throughout all six bars.

We hypothesized that targets with small PE_pred_s and high uncertainty produce highly variable and shorter saccade trigger times. As outlined in the pre-registration, PE_pred_ was calculated continuously for each saccade trial, sampled at target step, and averaged over a 50-ms window. Trials were then sorted into three bins of <–5°, –5 to 5°, and >5° PE_pred_ bins with their corresponding saccade trigger times. Large negative PE_pred_ was defined to be less than –5° and large positive PE_pred_ defined to be >5°. Small PE_pred_ was defined to be between –5° and 5°. Means and standard deviations were not compared as the underlying trigger time distributions were non-Gaussian, therefore the medians and IQRs were more informative. Trigger time distributions for each bin were plotted for clear and blurred conditions (Fig. 11*A–F*). Median trigger times and IQR for each PE_pred_ bin was calculated for each subject and compared across bins and conditions (Fig. 11*G*).

There was a significant effect as determined by repeated measures one-way ANOVA between PE_pred_ magnitude and both median trigger time (*F*_(2,14)_ = 219.292, η^2^_p_ = 0.940, *p* = 3.101e+14, BF_10_ = 1.190e+31) and IQR (*F*_(2,14)_ = 108.47, η^2^_p_ = 0.886, *p* = 5.6329e-8, BF_10_ = 5.230e+16). Confirming hypothesis 1, a *post hoc* multiple comparison with Bonferroni correction revealed that small PE_pred_ trigger times were significantly longer than large PE_pred_ trigger times (+∼72 ms, *p* = 1.2517e-9, 5.720e+9) and had a larger IQR (+∼47 ms, *p* = 1.0421e-7, BF_10_ = 2.676e+7) compared to both negative and positive large PE_pred_. There was no significant difference between median trigger time (*p* = 0.481, BF_10_ = 1.156) and IQR (*p* = 1.0, BF_10_ = 0.195) for negative and positive large PE_pred_ bins.

To observe the effects of a Gaussian blur on the trigger time distributions with respect to PE_pred_ bins, a repeated measures two-way ANOVA was used to compare median trigger time and IQR of the same PE_pred_ under clear and blurred target conditions. A Bayes two-way ANOVA was used to estimate the Bayes Factor. There was a statistically significant interaction between the effects of PE_pred_ bins and target condition on median trigger time (*F*_(2,28)_ = 30.043, η^2^_p_ = 0.682, *p* = 0.000081, BF_10_ = 1.190e+31) and IQR (*F*_(2,28)_ = 8.237, η^2^_p_ = 0.370, *p* = 0.012, BF_10_ = 8.989e+18). *Post hoc* multiple comparisons confirmed Hypothesis 3 and revealed a significant increase in median trigger time (+37 ms, *p* = 4.4253e-8, BF_10_ = 3.668e+7) and weakly significant increase in IQR (+7.2 ms, *p* = 0.029, BF_10_ = 0.85) for all PE_pred_ bins with the addition of a Gaussian blur. Uncertainty most strongly modulated the trigger time when PE_pred_ was small (+68 ms) but did not significantly increase the IQR, as shown in Figure 11.

The bimodality in Figure 11*B* results from two underlying distributions of early saccades and late saccades (cutoff of 175 ms) addressed later in this article. This bimodality largely disappears in Figure 11*E* under blurred conditions, where the majority of trials are late saccade trials.

### Pre-registered hypotheses 2 and 3

**Figure 12.**
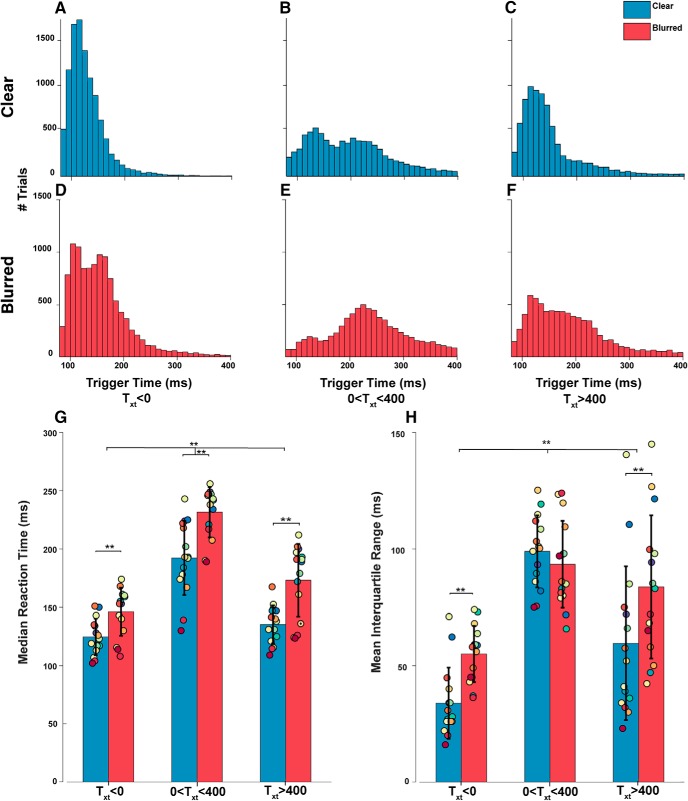
Saccade trigger time distributions and medians by T_xt_. Similarly to Figure 5, at target step, PE and RS can be approximated by PS and RS, and the index T_xt_ is used to estimate time to contact and directionality. Positive T_xt_ represents foveopetal motion and negative T_xt_ represents foveofugal motion. Trials were grouped into bins of negative (***A***, ***D***) small positive (***B***, ***E***), and large positive (***C***, ***F***) T_xt_. The bimodalities in ***B***, ***D***, ***E*** represent distributions of early and late saccades, which will be addressed later in this paper. ***G***, Collapsed median trigger times for all subjects for clear and blurred condition. ***H***, Collapsed IQR of all subjects for clear and blurred condition; ** represents a significant difference between groups with a *p* < 0.001. Each colored dot represents a particular subject’s mean value throughout all six bars.

We hypothesized that saccades made to targets moving toward the fovea, as measured by T_xt_, will have highly variable and longer trigger times. Trials were divided into bins of T_xt_ (−$\frac{Position\,\;step}{Velocity\,\;step})$with corresponding median trigger time and IQR for each subject and compared using repeated measures one-way ANOVA with *post hoc* comparison using Bonferroni correction, and a Bayes one-way ANOVA . The T_xt_ bins were <0 ms, 0–400 ms, >400 ms. Positive T_xt_ corresponds to foveopetal movement and negative T_xt_ corresponds to foveofugal movement. Similarly, trigger time distributions for each bin were plotted for clear and blurred conditions (Fig. 12*A–F*). Median trigger times and IQR for each PE_pred_ bin was calculated for each subject and compared across bins and conditions (Fig. 12*G*).

There was a significant effect as determined by repeated measures one-way ANOVA between median trigger time (*F*_(2,14)_ = 406.211, η^2^_p_ = 0.967, *p* = 9.6867e-12, BF_10_ = 296.752) and IQR (*F*_(2,14)_ = 34.663, η^2^_p_ = 0.712, *p* = 0.00004, BF_10_ = 1.692e+11). *Post hoc* multiple comparison with Bonferroni correction confirmed hypothesis 2 and revealed that trials with T_xt_ between 0 and 400 had significantly higher median trigger time (+76 ms, *p* = 9.8068e-12, BF_10_ = 1.783e+16; +57 ms, *p* = 7.3585e-12, BF_10_ = 5.359e+13) compared to negative T_xt_ and T_xt_ > 400, respectively, and significantly higher IQR (+51 ms, *p* = 2.0519e-8, BF_10_ = 1.492e+9; +25 ms, *p* = 0.036, BF_10_ = 21.32). Negative T_xt_ trials had a significantly lower median trigger time (–18 ms, *p* = 1.0e-5, BF_10_ = 593987) and IQR (–27 ms, *p* = 0.000316, BF_10_ = 135190.77) compared to trials with T_xt_ > 400. A two-way ANOVA revealed weak statistically significant interaction between the effects of T_xt_ bins and target condition on median trigger time (*F*_(2,28)_ = 5.070, η^2^_p_ = 0.266, *p* = 0.041, BF_10_ = 1.100e+33) and significant interaction effects for IQR (*F*_(2,28)_ = 11.329, η^2^_p_ = 0.447, *p* = 0.005, BF = 2.274e+13). *Post hoc* multiple comparisons revealed a significant increase in median trigger time (+32 ms, *p* = 6.6819e-7, BF_10_ = 2.656e+10) and IQR (+13 ms, *p* = 0.001, BF_10_ = 51.76) in the Gaussian blur condition.

### Bimodal trigger time distributions of early and late saccades

**Figure 13.**
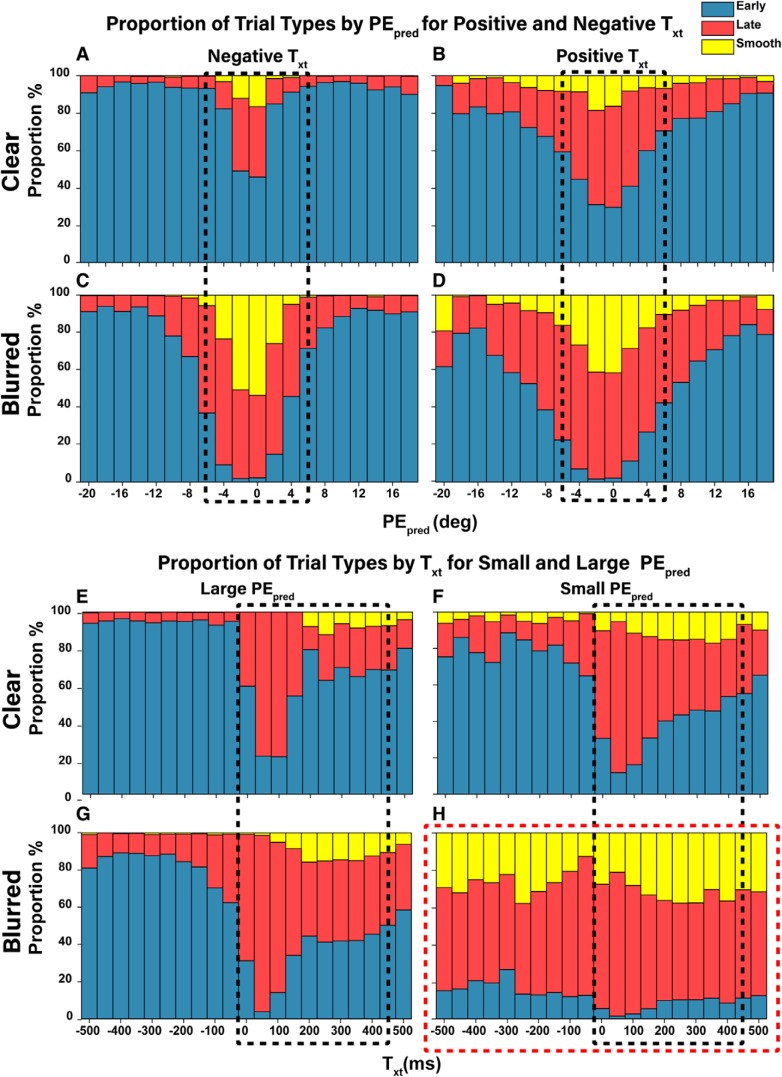
Proportion of early, late, and smooth trials under different PE_pred_ and T_xt_ conditions. Trials were classified as early, late, or smooth based on the trigger time cutoffs of 175 and 400 ms, as informed by trigger time distributions from Figures 5. ***A–D***, Variations of trial types by PE_pred_ bins under foveofugal and foveopetal T_xt_. ***E–H***, Variations of trial types by T_xt_ bins under small and large PE_pred_.

Thus far, both PE_pred_ and T_xt_ provide adequate explanations for the saccade trigger time, exhibiting similar distributions and effects in high uncertainty. This should come as no surprise as both metrics measure a property of target dynamics, future PE being measured by PE_pred_, while rate and direction of change of PE are captured by T_xt_. The bimodal distributions seen in both Figures 11*B*, 12*B* hint at two underlying distributions of early and late saccades, with a trigger time boundary of 175 ms. Under high uncertainty, a shift in favor of late saccades occurs. To explore why, we classified all trials into early, late, or smooth categories using the 175-ms boundary and plotted trial proportions under different PE_pred_ and T_xt_ conditions (Fig. 13).

These proportion plots explain the bimodality distributions in Figures 11, 12. In Figure 13*A*,*B*, when PE_pred_ was small (boxed region), the proportion of late trials was significantly higher when T_xt_ is positive compared to when it was negative. In Figure 11*B*, the early peak corresponds to Figure 13*A*, while the late peak corresponds to Figure 13*B*. Blurred target conditions expand this zone and shift the proportions to favor late saccades, especially when PE_pred_ was small (Fig. 13*C*,*D*, boxed region). Similarly, the 0- to 400-ms region in Figure 13*E*,*F* correspond to the bimodalities in Figure 12*B*. When PE_pred_ was small, the proportion of late trials was significantly higher compared to when PE_pred_ was large. Interestingly, under blurred conditions and small PE_pred_ (Fig. 13*H*), all T_xt_ values from negative to positive resulted in high proportions of late and smooth trials. This unintuitive break in the pattern serves as a strong indication that PE_pred_ rather than T_xt_ is used as a decision signal for the catch-up saccade trigger mechanism, although information captured by T_xt_ may still influence the rate of rise.

### PE_pred_ triggers catch-up saccades

**Figure 14.**
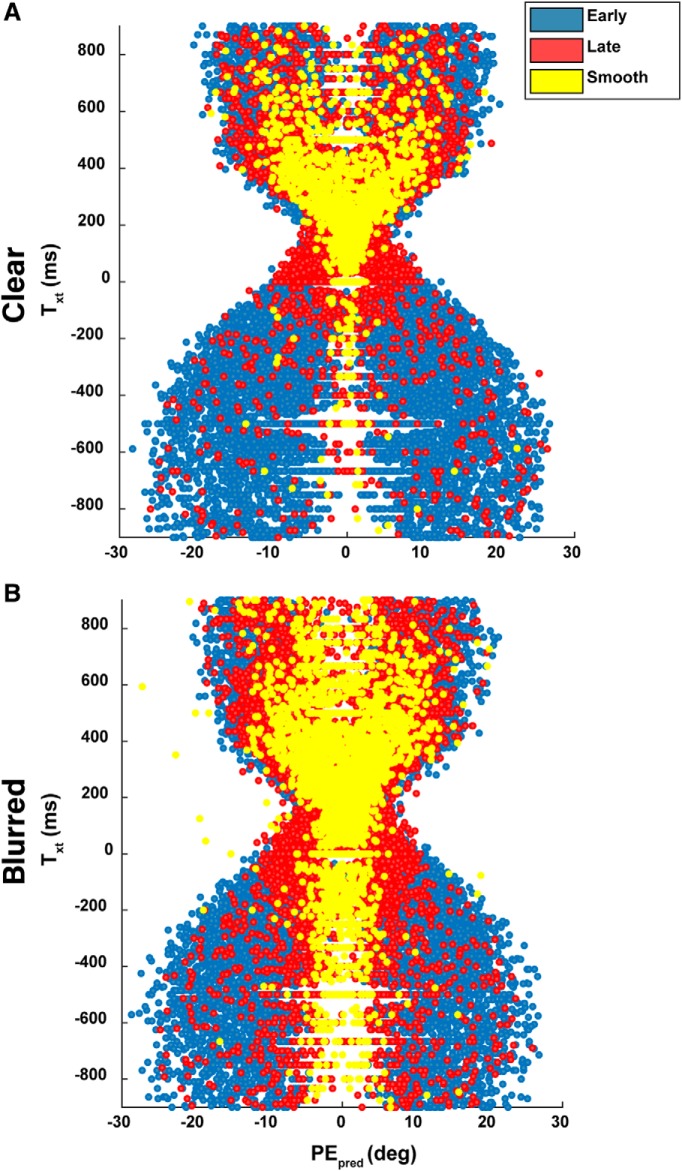
Individual trial scatterplot of PE_pred_ and T_xt_. To further illustrate the correlations between PE_pred_ and T_xt_, with saccade trigger times, single trials were plotted and assigned a color corresponding to their trigger time category. ***A***, Early saccades are evenly distributed along the parameter space, whereas smooth trials and late saccades are most evident for positive T_xt_ and small PE_pred_. ***B***, Blurred target conditions disperse late and smooth trial distributions as higher uncertainty reduces the likelihood for a saccade trigger.

Lastly, for each trial type, we sampled and plotted PE_pred_ and T_xt_ as a scatterplot (Fig. 14). Under clear conditions, the majority of late and smooth trials appeared at small PE_pred_ and positive T_xt_. There was a strong boundary at the 0 T_xt_, where the majority of smooth trials occur. When uncertainty was high however, the boundary disappeared and both late and smooth trials were dispersed along the T_xt_ axis. In contrast, in both target conditions, the 0 ± 10° boundary of the PE_pred_ axis remained. Trials outside the boundary were largely early saccade trials while those within the boundary were late or smooth saccade trials.

## Discussion

### General discussion

In this study, we investigated the trigger mechanism of catch-up saccades during sustained smooth pursuit. Our results provide strong support for the central role of PE_pred_ in the trigger mechanism. As previous studies have shown, PE and RS are the primary sensory inputs that drive the saccadic-pursuit system (Orban de Xivry and Lefèvre, 2007). Suppression of saccade triggering is more probable when PE naturally decays due to RS in the opposite direction. We have further demonstrated that noisy estimates of PE and RS are likely used to predict future PE and make a trigger decision. Trials that generated predicted errors below 5° frequently resulted in the suppression of saccade triggering or a triggering of a late saccade, whereas large predicted errors consistently triggered early saccades. Additionally, trigger time variability significantly increased when the preceding predicted error was small. By blurring the tracking target, we also demonstrated that high levels of sensory uncertainty strongly modulate the trigger decision. High uncertainty resulted in stronger saccade suppression when predicted errors were below 5° but had no effect when predicted errors were large. High uncertainty also resulted in an overall increase of saccade trigger times, most significantly when predicted errors were small, although the effects on trigger time variability were weak. Saccades to targets moving foveopetally also resulted in longer and more variable trigger times, consistent with previous work (Bieg et al., 2015). Lastly, we observed significant trigger time increases due to both SDN from large RS and noise from high sensory uncertainty, further supporting a probabilistic explanation for the trigger decision. For each observation, the explanatory power of PE_pred_ was compared to the previously suggested metric of T_xe_ or T_xt_ (de Brouwer et al., 2002b). Overall, if in doubt of sensory inputs, the saccadic system suppresses the triggering of a catch-up saccade in favor of smooth pursuit. In comparison to T_xe_s, explanations of behavioral data using PE_pred_ were more stable, intuitive, aligned with current literature (for review, see Shadmehr et al., 2010), and easily translatable to 2D or 3D tracking tasks where targets may not cross the initial point of fixation.

**Figure 15.**
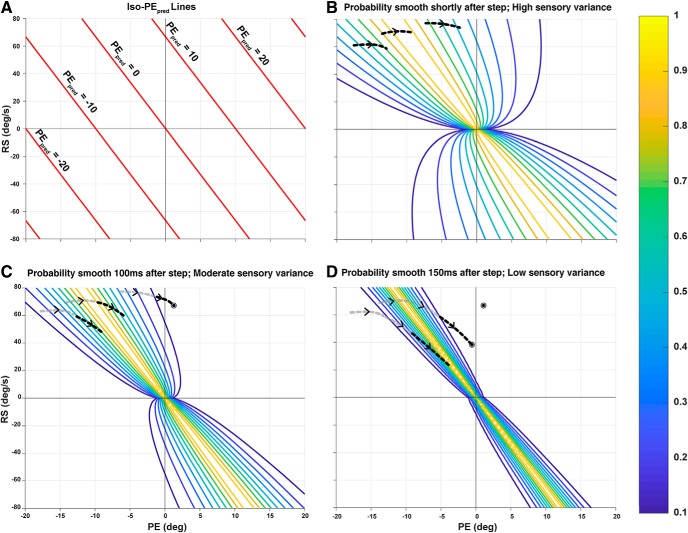
Illustration of a dynamic smooth zone. Visual illustration of how the width of the smooth zone or the decision boundary to trigger a saccade constricts as the variance of sensory estimation decreases over time. ***A***, Iso-PE_pred_ lines on a phase plot where PE_pred_ remains the same. Saccades are suppressed when predicted error is around 0. ***B***, Probability lines around PE_pred_ = 0 shortly after the step where subjects should smoothly pursue the target without needing to trigger a saccade. This is where variance of sensory estimation of RS from Kalman filtering is highest. Three hypothetical temporal evolutions are shown as a visual aid. ***C***, Probability lines narrow slightly as evidence is accumulated and variance decreases. One trial exits the smooth zone and a saccade is triggered. ***D***, Probability lines are very narrow as the variance around RS estimates is small. Saccades will be triggered shortly after exiting this narrow zone. Another trial exits the smooth zone and a saccade is triggered. The remaining trial is still in smooth pursuit.

The smooth zone, as defined by T_xe_, is tightly linked to our proposed predicted error mechanism. For example, consider Equation 1 in a situation when PE_pred_ is 0 and a saccade is not needed. Rearranging this equation to solve for dt returns Equation 3 for T_xe_. In fact, our chosen dt for linear extrapolation is 150 ms, which is within the T_xe_ smooth zone. However, T_xe_ is a deterministic measure and does not include measures of uncertainty. Predicted error, however, accounts for uncertainty through the variance of the Kalman filtering estimate. At sudden stimulus changes, the variance of RS and PE is high; therefore, the probability distribution of having 0 predicted error is broad. As the variance decreases over time with better estimates, so does this distribution constrict around the true PE_pred_ = 0 value (Fig. 15).

On the phase plot, a trial in the smooth zone can either move in the direction of the constricting boundary or opposite to it. In the former, the subject remains in the smooth zone for longer while in the latter, a participant exits the zone faster and triggers a saccade, resulting in high density saccade regions on either side of the 0 PE boundary (Fig. 7). In this view, the effect of the blurred target condition will lead to higher position uncertainty and a wider decision boundary which result in higher saccade trigger times and smooth trials.

### Limitations and alternative interpretations

An alternative interpretation of our results from the blurred target conditions is that participants were simply more tolerant of small PEs when the target was larger, as they could direct their gaze to a larger area. This is likely not true as the qualitative trends in both the clear and blurred target conditions are in agreement, and the observed quantitative differences match our hypothesized results. Another weakness in most eye-tracking studies, including this one, is that laboratory tracking tasks show little resemblance to tracking in the natural world. For example, the use of small foveal targets, uncommon in nature, may elicit saccades more frequently (Heinen et al., 2016). Accordingly, larger stimuli such as our blurred target may increase the tolerance for PEs rather than the sensory uncertainty, thus suppressing saccades and driving smooth pursuit. However, Melcher and Kowler (1999) demonstrated that regardless of stimulus shape, the saccadic landing position is computed as the center-of-area of that stimulus. Therefore, it is highly likely that for a given target location, the PE_pred_ for small or large targets will be the same.

The observed effects of saccade trigger times under different magnitudes of predicted errors could also partly be explained by whether attention allocation is ahead, or broadly ahead of a moving target (Khan et al., 2010; Chen et al., 2017), as attention will modulate the saccade trigger times. Additionally, a confound in our study is the heterogenous tracking ability of participants. Indeed, smooth pursuit performance predicts interception accuracy, strategy, and whether interception is early or late (Fooken et al., 2016). Thus, subjects who are naturally better at smooth pursuit such as those who play ball sports or video games may better correct for PEs using pursuit alone. Although some subjects were better smooth pursuers, we observed similar trends across all subjects when results were plotted individually, in agreement with our overall hypotheses.

Furthermore, our results from Figures 13, 14 suggest that T_xt_ is still relevant as it indicates whether predicted errors will continue to increase or decrease. Chang and Jazayeri (2018) show that humans indeed exploit temporal information to make interception decisions. Perhaps humans combine information from both predicted errors and T_xt_ to make the trigger decision. Lastly, some suggest that the superior colliculus is not involved in prediction as no neural correlates have been observed (Goffart et al., 2017). However, perhaps prediction is computed elsewhere in the brain and the necessary saccade amplitude is added to the superior colliculus motor command. Such mechanism would explain the asynchrony observed in the position and motion pathways of the saccadic system (Schreiber et al., 2006). Indeed, (Keller et al., 1996) observed systematic shifts in the movement fields of SC neurons when saccades were made to moving targets, concluding that an extrapolation pathway that may not include the SC may explain these observations.

### Comparison to literature

#### Separate versus interacting systems

Until recently, saccades and pursuit have been separately characterized both functionally and anatomically. However, the past two decades of research have demonstrated that these two types of eye movements may originate from a single sensorimotor process (Krauzlis, 2004; Orban de Xivry and Lefèvre, 2007). Behavioral interactions between the saccadic and smooth pursuit system have been extensively investigated. The two systems are driven by both PE and RS inputs slip (Keller and Johnsen, 1990; de Brouwer et al., 2002b; Blohm et al., 2005; Etchells et al., 2010), supporting the use of both sensory estimates in the preparation of catch-up saccades. Moreover, both systems share a common predictive process mediated through the supplementary eye field (SEF; Nyffeler et al., 2008) and exhibit similar dependence on movement preparation time (Joiner and Shelhamer, 2006). The two systems are also intertwined anatomically and functionally. Frontal eye field (FEF) and SEF, LIP, and large brainstem and cerebellar pathways have both saccade and pursuit related activity (for review, see Orban de Xivry and Lefèvre, 2007). We further provide support for the hypothesis that the inputs to the saccadic and pursuit system are intertwined and used in combination to compute predictions of future PEs, and that these predictions are modulated by sensory uncertainty.

#### Saccade triggering

The LATER model accurately reproduces the trigger time distributions of saccades triggered to stationary targets observed in human participants (Noorani and Carpenter, 2016). However, the model does not account for the sensorimotor context such as target directions, pursuit gain, or RS. As discussed, saccades and pursuit are highly interacting systems. Krauzlis and Miles (1996) demonstrated that saccades and pursuit share the same inputs for the release of fixation from stationary targets on movement initiation. Perhaps the same is true for catch-up saccade triggering during smooth pursuit. Saccade trigger times to moving targets appear asymmetrical, as targets moving away from the fovea are triggered faster both during pursuit initiation and ongoing pursuit (de Brouwer et al., 2002a; Bieg et al., 2015). These trends are adequately captured by the accumulated confidence in predicted error model, while the presented behavioral results provide further evidence for a predictive and uncertainty dependent trigger mechanism as seen by saccade occurrence and trigger times. Thus, it appears that catch-up saccades are another outcome of a singular sensorimotor process that governs both smooth pursuit and saccadic eye movements.

#### Decision making

Visual and motor latencies account for ∼50% of the length and variability of saccade trigger times (Gold and Shadlen, 2007), suggesting a more complex decision process. Statistical decision making is an inference process whereby prior beliefs, incoming evidence, and time constraints are combined by a decision maker to choose between two or more competing hypotheses about the state of the world. Models of motor planning based on this theory use uncertain evidence and explicit action penalties and successfully predict human behavior (Trommershäuser et al., 2003). In the context of this study, the decision is to trigger or suppress a saccade. We hypothesized that confidence in future error estimates are used as a decision signal to coordinate the triggering of a saccade or to remain in smooth pursuit. signal detection theory (SDT), a common decision-making framework, incorporates a probability ratio of distributions representing parameters, observations, or competing hypotheses (Gold and Shadlen, 2007). The confidence in a particular decision is defined as the probability that a decision is correct given the evidence (Pouget et al., 2016). An extension of SDT is the sequential probability ratio test (SPRT), where sequential analysis of incoming samples accumulate over time to reach a positive or negative decision bound (Wald and Wolfowitz, 1948). Given a certain decision threshold, we expected to observe differences in trigger times based on the proposed decision mechanism. That is, a quicker rise to threshold given large or sustained predicted errors and high confidence in those estimates. Our results match our expectations since predicted error magnitudes before saccades inform whether a saccade will occur and what the trigger time will be. This is consistent with previous work that saccades and pursuit share a common decision signal but use different thresholds and are subject to different delays (Liston and Krauzlis, 2005). Thus, the motor control and coordination of saccades and smooth pursuit is viewed as a decision-making process.

#### Implications for models

When motor control in the brain is viewed as a decision-making process, it can be modeled using decision boundaries and evidence accumulation. Behavioral, neurophysiological, and computational research has revealed many links between decision-making processes and sensorimotor control (for review, see Wolpert and Landy, 2012; Gallivan et al., 2018). Models of decision making such as the LATER model have accurately predicted reaction time distributions for complex psychophysics tasks such as Go-NoGo, countermanding, and tasks where continuous evaluation of new sensory evidence is required (Noorani and Carpenter, 2016). In particular, the superior colliculus has been suggested to implement a decision process before the preparation of saccades (Ratcliff et al., 2003; Lo and Wang, 2006). Our model suggests a similar decision process of evidence accumulation takes place for the saccade trigger mechanism (Coutinho et al., 2018). The model predictions of saccade trigger times distributions are consistent with the behavioral results observed in this study. Furthermore, both our model and behavioral data provide new insights into how uncertainty in sensory inputs modulate oculomotor responses during tracking behavior. Our results imply that models must take into account information regarding prediction of future errors, crossing time information, and sensory noise to be able to account for all possible trigger behaviors.

### Motor control and uncertainty

Neural control of movement is limited by noise from both sensory inputs such as vision and proprioception and from motor commands. However, the majority of motor variation in eye movements (∼92%) can be attributed to sensory noise such as errors of target motion or position estimates (Osborne et al., 2005). At the cognitive level we refer to this noise as uncertainty, which greatly influences our motor decisions. The central nervous system evolved to minimize the impact of sensory uncertainty by integrating information from numerous modalities and waiting to accumulate more information (van Beers et al., 2002). We proposed that uncertainty similarly modulates the catch-up saccade trigger mechanism, whereby highly uncertain stimuli such as a blurred or fast target will require further evidence accumulation and result in later or suppressed saccades. Similar perception-action tradeoffs in a ball-interception task under sensory uncertainty and time constraint are near Bayes-optimal (Faisal and Wolpert, 2009). Our model extends an existing Bayesian model of motion estimation and smooth pursuit using Kalman filtering (Orban de Xivry et al., 2013) with the addition of a future error estimation and a decision pathway for evidence accumulation. In the face of uncertainty, near-optimal Bayesian behavior has been previously observed in hand movement dynamics as well as perception (Stocker and Simoncelli, 2006; Acerbi et al., 2017) and a neural implementation of Bayesian estimation has been exhibited in the FEF during smooth pursuit (Darlington et al., 2018). Our model-guided behavioral results of how uncertainty modulates the saccade trigger decision are consistent with a Bayesian observer that optimally combines sensory inputs and their uncertainties to produce near optimal behavior. We further provide insight that the trigger mechanism likely relies on a probabilistic estimate of PE_pred_s through linear motion extrapolation.

### Underlying neurophysiology for prediction

Motion extrapolation provides a useful mechanism for the brain to make sense of a continuously changing sensory environment. For example, catch-up saccades programming involves extrapolation of target position using current target velocity to land on the target by the time of saccade execution (de Brouwer et al., 2001; Lisi and Cavanagh, 2017). The FEFs were proposed to create a prediction map of stable visual percepts able to report and correct prediction errors following a saccade and were also correlated with saccadic trigger time (Connolly et al., 2005; Crapse and Sommer, 2008). Internal models of target motion are necessary to overcome visual delays inherent to the visuomotor system but also for instances of target occlusion and interceptive movements (Becker and Fuchs, 1985; Bosco et al., 2015). These internal models are useful as they allow the brain to minimize prediction error and behave optimally in the environment (McNamee and Wolpert, 2019). Notably, the eye position signals decoded from visual areas such as MT, MST, VIP, and LIP lead the current eye position, indicating a predictive process based on an efference copy of eye position (Dowiasch et al., 2016). This efference copy likely originates from area MST, where neurons account for effects of eye movements and can be used to inform saccade planning (Tanaka, 2005). Thus, it is highly plausible and supported by our results that prediction error plays a central role in the coordination of saccades during smooth pursuit. We observed that humans predict future errors by extrapolating 150 ms in the future, consistent with similar time estimates for smooth pursuit and memory guided saccades (Diaz et al., 2013; Orban de Xivry et al., 2013). Due to the heavily intertwined saccade and pursuit pathways, several brain areas are likely candidates to compute this prediction, such as the FEF/SEF or PPC. Other potential sites where extrapolation could occur by integrating position and velocity signals are the nucleus reticularis tegmenti pontis (NRTP) and the vermis cerebellum, as suggested by Schreiber et al. (2006). While previously suggested explanations for the saccade trigger such as T_xe_ or T_xt_ do not provide a simple mechanism, they remain behaviorally relevant. As shown, negative or positive crossing time indicates an increasing or decreasing PE_pred_, respectively. Although predicted error trigger times distributions are centered on zero, positive or negative T_xt_ can widen or narrow the distribution.

### Conclusion

In conclusion, we show that PE_pred_ and uncertainty in sensory estimates are used to make catch-up saccade trigger decisions, and that they adequately explain saccade trigger times and behavioral variability to abrupt changes of position and VSs. Our study complements a breadth of literature in support of uncertain motor decision-making and predictive behavior. to further investigate this decision mechanism, acceleration inputs should be considered as they are known to modulate the smooth pursuit response (Brostek et al., 2017). Effects of predicted errors and uncertainty on saccade programming of amplitude may also be apparent on closer examination. Additionally, adapting the double-step ramp task to more naturalistic tracking behavior may be necessary to reinforce the brain’s use of this trigger mechanism. Models of saccade and pursuit should collapse previously independent pathways known to be shared by both systems and integrate a component of sensory uncertainty. Finally, a focus on the neural correlates of sensory prediction and/or confidence as well as where the trigger decision is made will serve to strengthen arguments for this proposed mechanism.

## Synthesis

Reviewing Editor: Satu Palva, University of Helsinki Decisions are customarily a result of the Reviewing Editor and the peer reviewers coming together and discussing their recommendations until a consensus is reached. When revisions are invited, a fact-based synthesis statement explaining their decision and outlining what is needed to prepare a revision will be listed below. The following reviewer(s) agreed to reveal their identity: Martha Havenith, Christian Keitel.

Statistics

Effect sizes (eta- or omega-squared) should be added when reporting (frequentist) ANOVA results.

Synthesis

In their manuscript 'Predicted position error triggers catch-up saccades during sustained smooth pursuit', the authors present recordings of human saccade behaviour during smooth pursuit of moving visual targets. Their analyses reveal that saccades are predicted by the size and certainty of the extrapolated future deviation between target position and foveal position, rather than by the size of the current deviation. As a result, larger uncertainty about the future target position (introduced by blurring the target) results in lower saccade probability. These findings align the process by which saccades are triggered during smooth eye movements with a more general framework of decision making in uncertain conditions. The study makes a strong case why saccades during smooth eye movements are triggered by the predicted future (rather than the current) deviation between eye and target position. This aligns the process of saccade triggering with a more general conceptual framework of decision making in the presence of uncertainty. The study was clearly designed and the data analysis thorough. Statistical analysis and organization of the manuscript would, however, be improvement.

Major comment

Statistical analyses were carried out with ANOVA. Effect sizes should be added when reporting ANOVA results.

The rationale for the study is explained in the discussion but not in the introduction. The concepts and rationale of the study should already be explained in the introduction, so that readers would have a better framework to interpret the subsequent results.

The reviewers found the current structure of results confusing. Please try to clarify the text.

In the discussion, the authors mention that the blurred target is effectively larger than the clear one, and there is a confound in terms of stimulus size. We suggest that authors could add, if possible, a control experiment with a few subjects in order to address this potential confound explicitly. If not possible, please discuss this putative confound in the discussion.

Minor

For instance, if I understand this correctly, one big difference to previous work is that saccade triggers are based on predicted rather than actual position errors. For me, the second take-away was that, if in doubt, the system suppresses saccades. I would reiterate this more strongly throughout introduction and discussion. Similarly, the comparison with previous metrics is worth emphasizing already in the introduction: Mention that 'eye crossing time' is the classical measure that's thought to predict saccades, that there are reasons to believe this is not ideal (it makes sense to have uncertainty as a factor, a purely deterministic system is unlikely to work well...), and that in order to test this idea you are examining both metrics (eye crossing time and predicted position error) throughout the whole study.

Some specific applications of that general rationale:

o Line 62: Here you can add the arguments you only mention in the discussion right now - that it makes sense to have uncertainty as a factor, a purely deterministic system is unlikely to work well etc.

o Page 4: In the discussion, you mention that your approach is an extension of the LATER model. If so, would be good to mention this here and explain in which way. (Otherwise rewrite that bit of the discussion please)

o Page 6: Explain basis for your hypotheses briefly: That uncertainty matters, that saccade initiation follows similar rules as other types of decision making.

o Line 100: Add a bit more rationale to the null hypothesis as well: Make explicit the difference between previous models and your model by mentioning eye-crossing time again. So the null hypothesis would be something like 'Saccades are triggered only by the time to foveation, not by predicted position error, with a small time to foveation suppressing saccades.'

o Line 695 - I would already mention this reasoning in the introduction to explain why you predict the predicted position error to be a better determinant of saccades

o Around line 680: Say explicitly that if in doubt, the system suppresses saccades

• Is there any reason why positive versus negative values of the PEpred (and RS) matter? In the data, it seems that there is no difference (see e.g. Fig. 11), and I also can't come up with a conceptual reason (leftwards versus rightward position errors should cause the same problem to the visual system right?). If this is correct, I would redo figures with the absolute size of PEpred, because it makes its relation to other variables much clearer. See below for details.

o Figure 7: Since you need positive and negative PEpred in order to show example trajectories properly, I would show example trajectories separately and add scatter plots of all trials using the absolute values of PRpred and RS, because that'll make much more obvious in which parameter space RS and PE tend to move right before a saccade.

o Figure 8: Same as Fig. 7 - you would see an instant relationship, where you essentially have an asymptotic curve for smooth pursuits and anything outside that area results in a saccade

o Figure 9: Same - absolut PEpred would show the relationship more clearly

o Figure 11/Line 529: I'd use absolute PEpred and then form only two categories (small versus large absolute PEpred). That makes the histograms more obvious.

o Figure 13 A-D Again would definitely collapse PEpred to absolute values. Makes plots much easier to read (and I don't see any asymmetries that would preclude using absolute values). Also would say foveopetal/fugal instead of positive negative??

o Figure 14 I'd again put abs of PEpred, and then integrate the figure with figure 13 because it's basically just a different way of showing the same thing?

• Page 13: Testing Hypothesis 1: Is there a reason why you don't use (rank) correlation of all data points instead of binning them? I'd say (by definition somewhat arbitrary) bins are always better avoided if possible. That said, the data seem very clear, so I don't believe this will change the ultimate outcome of the analyses.

• Figure 11: Have you tried drawing lines between individual subjects instead of/in addition to dot colors? If that works well, it would give people a chance to see how consistent the effect is across subjects.

• Figure 11: I would add a panel showing only IQRs across conditions

• Line 739: Concerning consistency of the shown effects across subjects irrespective of smooth-pursuit ability, an additional figure panel with a couple of individual traces to support this claim would be great.

Writing/editing comments

Line 26: you don't need the 'additional' since you are already 'adding'

Line 62: Mention that this is the 'eye crossing time' so that people can connect the dots that later.

Line 90: Get rid of the words in parentheses. It makes the reading of the hypotheses harder, and one naturally assumes that if greater x leads to great y, smaller x also leads to smaller y etc. Also, some text seems to be in bold, and I don't understand why, so I would just put everything in normal font.

Line 185: I would move the variable explanations from line 193 up to before the equations. That makes it easier to understand the equations afterwards.

Line 193 Why is this in bold font?

Line 451: This sentence is a bit confusing. Do you mean 'trials that either briefly cross or even remain in the smooth zone nevertheless trigger a saccade'?

Figure 12F: On the x axis I would put T>400 rather than 400<T

Line 616: I think you mean 'underlying'?

Line 688: Name examples of where/how crossing time is worse in the data

Figure 13 A-D: I would refer to foveopetal/fugal instead of positive/negative

Fig 15a+b: Colour bar seems to be partially covered by a figure background.

Line 844: How have you extended the model?

## Author Response

Predicted tracking error triggers catch-up saccades during smooth pursuit

Point-by-Point Reply

Thank you for your thoughtful comments and suggestions regarding this manuscript. We have worked to address each of your concerns and are pleased to submit a revised manuscript with the following revisions:

1. “Statistical analyses were carried out with ANOVA. Effect sizes should be added when reporting ANOVA results.”

Each reported ANOVA analysis now includes an partial eta squared effect size (η2p )

2. “The concepts and rationale of the study should already be explained in the introduction, so that readers would have a better framework to interpret the subsequent results.”

Additional sentences have been added to the introduction to more clearly explain the rationale for the upcoming hypotheses.

3. “The reviewers found the current structure of results confusing. Please try to clarify the text. ”

With regards to the order of the tested hypotheses, we chose this presentation order to make the manuscript logically self-contained and easy to follow. We would like to maintain this structure. We have ensured that tests of each hypothesis are clearly demarcated.

4. “In the discussion, the authors mention that the blurred target is effectively larger than the clear one, and there is a confound in terms of stimulus size. We suggest that authors could add, if possible, a control experiment with a few subjects in order to address this potential confound explicitly. If not possible, please discuss this putative confound in the discussion. ”

Though it is not possible for us to collect more data due to the time constraints of a graduate degree, we have added an additional sentence in the discussion to address this potential confound.

“However, Melcher and Keller (1999) demonstrated that regardless of stimulus shape, the saccadic landing position is computed as the center-of-area of that stimulus. Therefore, it is highly likely that for a given target location, the predicted position error for small or large targets will be the same.”

Nevertheless, as saccades have been shown to be modified by target size, the cofound in our results remains and is therefore addressed in the discussion to allow the reader to consider alternative explanations.

5. “. For me, the second take-away was that, if in doubt, the system suppresses saccades. I would reiterate this more strongly throughout introduction and discussion. Similarly, the comparison with previous metrics is worth emphasizing already in the introduction: Mention that 'eye crossing time' is the classical measure that's thought to predict saccades, that there are reasons to believe this is not ideal (it makes sense to have uncertainty as a factor, a purely deterministic system is unlikely to work well...), and that in order to test this idea you are examining both metrics (eye crossing time and predicted position error) throughout the whole study.”

This suggestion and the following specific applications mentioned for lines 64, 100, 695, 680 page 4, page 6, as per the reviewers comments, have now been addressed in both the introduction and discussion to re-iterate the bottom line of our proposed hypotheses and how the following manuscript will compare eye-crossing time and predicted position error

6. “Is there any reason why positive versus negative values of the PEpred (and RS) matter? In the data, it seems that there is no difference (see e.g. Fig. 11), and I also can't come up with a conceptual reason (leftwards versus rightward position errors should cause the same problem to the visual system right?). If this is correct, I would redo figures with the absolute size of PEpred, because it makes its relation to other variables much clearer. See below for details.”

We have chosen to show both positive and negative instances of PEpred to ensure there are no hidden asymmetries in the data. The reviewers are correct that, conceptually, there should be no difference between leftward and rightward errors. However, in order to show readers that we are not masking any asymmetries by taking the absolute PEpred, we chose to show all results for both positive and negative predicted errors.

7. “Figure 7: Since you need positive and negative PEpred in order to show example trajectories properly, I would show example trajectories separately and add scatter plots of all trials using the absolute values of PRpred and RS, because that'll make much more obvious in which parameter space RS and PE tend to move right before a saccade. ”

We have tested this suggestion and produced the following figure:

In this figure, it is much more difficult to interpret the sensory information being used to compute the trigger decision. Information is lost pertaining to relative target direction instantaneous sensory inputs. Since our proposed mechanism for the saccade trigger heavily relies on the conceptual understanding of the variety of possible sensory inputs that feed into the saccadic and pursuit system, we elected to represent the phase plot in terms of position error (PE) and retinal slip (RS) instead of absolute predicted position error, which is more abstract and difficult to conceptualize.

This explanation, as well as the one provided in point 6 address several reviewer comments regarding Figures 7-14 about the use of abs(PEpred) rather than PEpred.

8. Page 13: Testing Hypothesis 1: Is there a reason why you don't use (rank) correlation of all data points instead of binning them? I'd say (by definition somewhat arbitrary) bins are always better avoided if possible. That said, the data seem very clear, so I don't believe this will change the ultimate outcome of the analyses.

Although rank correlation is more appropriate, due to the constraints and nature of a pre-registered report, we cannot change the proposed analysis that addressed the pre-registered hypothesis. As stated by the reviewer, we do not believe this to be an issue as the data is very clear about the observed effects.

9. Figure 11: Have you tried drawing lines between individual subjects instead of/in addition to dot colors? If that works well, it would give people a chance to see how consistent the effect is across subjects.

Individual lines were included in an earlier version of the figure but were too distracted as there are 15 subjects and multiple comparisons between the three different bars in both conditions. We have added the coloured dots to show the spread of subjects and in case a reader may want to observe changes in a single subject

10. Figure 11: I would add a panel showing only IQRs across conditions

Figures 11 and 12 now include a separate panel to show the IQRs.

11. Line 739: Concerning consistency of the shown effects across subjects irrespective of smooth-pursuit ability, an additional figure panel with a couple of individual traces to support this claim would be great.

We have added a figure to show that there is no clear relationship between a participants' pursuit ability and their saccade trigger behaviour. We compared the average pursuit gain (eye velocity / target velocity) in the first motion ramp, with the difference in reaction times between large and small predicted errors for each subject. We expected to see no clear relationship between the two variables, confirming our claim that the shown effects of PEpred on reaction time are independent on pursuit ability.

The remainder of the writing and editing comments have been addressed and changed directly in the manuscript. We have also added sample data to the online GitHub repository as the full dataset can only be released by Dryad once the paper is published.

Thank you for your consideration

Omri Nachmani, Jonathan Coutinho, Aarlenne Khan, Philippe Lefèvre, and Gunnar Blohm.

References:

Melcher D, Kowler E (1999) Shapes, surfaces, and saccades. Vision Res 39:2929-46.
